# Assessment of *Clarias gariepinus* as a biological control agent against mosquito larvae

**DOI:** 10.1186/s12898-016-0081-0

**Published:** 2016-05-31

**Authors:** Buze Chala, Berhanu Erko, Abebe Animut, Abraham Degarege, Beyene Petros

**Affiliations:** Arba Minch University, P. O. Box 21, Arba Minch, Ethiopia; Aklilu Lemma Institute of Pathobiology, Addis Ababa University, P. O. Box 1176, Addis Ababa, Ethiopia; Department of Epidemiology, Robert Stempel College of Public Health and Social Work, Florida International University, Miami, FL USA; Microbial, Cellular and Molecular Biology Department, College of Natural Sciences, Addis Ababa University, P. O. Box 1176, Addis Ababa, Ethiopia

**Keywords:** *Clarias gariepinus*, Biological control, Larvae, *Anopheles arabiensis*, *Culex*, Ethiopia

## Abstract

**Background:**

The emergence and spread of insecticide resistant mosquitoes renewed interest in investigating the use of larvivorous fish as a biological control agent. The potential of *Clarias gariepinus* fish in controlling *Anopheles arabiensis* and culicine larvae was assessed under laboratory and semi-field conditions.

**Results:**

Small size (15–20 cm) *C. gariepinus* fish consumed greater number of mosquito larvae than the large size fish (25–40 cm) in the multivariate regression model (β = 13.36, 95 % CI = 4.57, 22.15). The *Anopheles* larvae consumed was greater in number than the culicines larvae consumed by the fish (β = 12.10, 95 % CI = 3.31, 20.89). The number of larvae consumed was greater during the night hours than during the light hours (β = 30.06, 95 % CI = 21.27, 38.85). Amount of supplementary fish food did not cause significant differences in the number of mosquito larvae consumed by the fish among different groups. *C. gariepinus* was observed to feed on mosquito larvae under laboratory and semi-field conditions.

**Conclusion:**

*C. gariepinus* fed on the larvae of *An. arabiensis* and culicines readily. Hence, it can be used as an alternative mosquito control agent in Ethiopia where the breeding habitats are small and localized.

## Background

Current malaria vector control strategies depend mainly on long-lasting insecticidal nets (LLINs) and indoor residual spraying (IRS). These interventions contributed to the reduction of malaria-related cases and deaths globally. However, the gains are threatened due to the emergence and spread of vectors that are physiologically and behaviourally resistant to the insecticides [1–3].

Besides the development of insecticide resistance in malaria vectors, increased concern of environmental pollution with insecticides have aroused interest in developing environment-friendly approaches such as the use of biological control agents [4, 5] including larvivorous fishes.

Over the past 100 years, several fish species have been documented to feed on mosquito larvae in different parts of the world among which many are proved effective [6, 7]. One of these species is *Clarias gariepinus* [7–9]. It is an opportunistic feeder targeting insects, worms, mollusks, gastropods, crustaceans, small fishes, aquatic plants and debris that can fit into its mouth [8]. In an effort to control mosquito-borne diseases, targeting the immature stages of mosquitoes (eggs, larvae and pupae) can be more effective as these stages are confined within relatively small aquatic habitats, relatively immobile and cannot readily escape the fish. In Ethiopia, *C. gariepinus* occurs almost in all water bodies that can support fish [10]. However, the efficacy of this fish in devouring mosquito larvae is poorly understood. In the present study, we assessed the efficacy of *C. gariepinu* as a biological control agent against mosquito (*Anopheles* and *Culex*) larvae in laboratory and semi-field conditions.

## Methods

### *Clarias gariepinus* fish

A colony of *C. gariepinus* fish and its supplementary food was obtained from the Sebeta National and Other Living Aquatic Resources Research Center (NFLARRC) of Ethiopia, near Sebeta Town, located at 20 km on the road to Jimma Town. The fish was maintained in glass aquaria (60 × 40 × 35 cm) and acclimatized at room temperature (25 ± 2 °C) before the experiment began. Aquaria air pumps were used to aerate the water in the aquaria.

### *Anopheles* and *Culex* larvae

*Anopheles arabiensis* larvae used in the experiment was obtained from the Aklilu Lemma Institute of Pathobiology (ALIPB) insectary whereas culicine larvae were obtained from the ponds of the ALIPB.

### Laboratory based mosquito predation experiment

Nine clean glass aquaria (each 40 × 20 × 26 cm), each filled with 15 L of aged tap water, were arranged in three groups. A total of 50, 100 or 150 *An. arabiensis/*culicine larvae were introduced into the 1st, 2nd and 3rd in triplicates. The first group was supplied with adequate supplementary fish food, the second with inadequate food and the third with no food. Inadequate food is supplementary food given for each *C. gariepinus*, which was about 2 % of each fish body weight while adequate food was about 4 % of each fish body weight as previously used [11]. Either small (15–20 cm) or large (25–40 cm) fish was placed into the first, second and third (control) groups. The experiment was conducted in the day light (12 h) and night dark hours (12 h) to compare larval predation by the catfish. The experiment for the day light hour started in the morning (6:00 am) and larval predation by the fish was recorded in the early evening (6:00 pm). The experiment for the day night hour was started in the early evening (6:00 pm) and larval predation by the fish was recorded in the early morning (6:00 am).

### Semi-field based *Anopheles arabiensis* larvae predation experiment

Semi-field larval predation experiment was carried out in four ponds of equal size (1.05 m × 0.40 m × 1 cm) built on the premises of the ALIPB. The ponds were washed and covered with mosquito proof net before and after introducing catfish and mosquito larvae. This was done to prevent escape of emerging adult mosquitoes and incoming egg laying mosquitoes. About 0.21 m^3^ of water and 300 *Anophelesarabiensis* larvae were introduced into each of the four clean ponds. The ponds were then categorized as group 1, 2, 3 and 4. Following the grouping, a small size fish (15–20 cm), a medium size fish (20–25 cm) and a large size fish (25–40 cm) were introduced into the 1st, 2nd and 3rd group, respectively. No fish was introduced into the 4th group (control). Supplementary fish food was added in each of the experimental groups, but not in the control.

### Data analysis

Data were double entered in excel-sheet and analyzed using STATA version 11 (STATA Corporation, College Station, Texas, USA). Percentage of mosquito larvae consumed was calculated by dividing the number of mosquito larvae consumed by the number of mosquito larvae exposed. Z test was used to compare the percentage of mosquito (anopheline or culicines) larvae consumed by small and large sized fish during night and light hours. Multivariable regression analysis was used to quantify the impact of fish size, exposure hour (day light hour and night hour), mosquito genus (*Anopheles* or *Culex*), number of mosquito larvae exposed and amount of supplementary food added on the feeding efficacy of the fish. 95 % CI values were calculated for the average mean difference of mosquito larvae consumed between different groups. Values were considered significant when p < 0.05 or when 95 % CI values did not include zero.

## Results

### Feeding activity of *Clarias gariepinus* on *Anopheles arabiensis* larvae in the laboratory

*Clarias gariepinus* was found to feed on most mosquito larvae it encountered both in the laboratory and in the semi-field conditions. Small size *C. gariepinus* consumed most of the *An. arabiensis* larvae it encountered and the night hour consumption (96.9 %) was significantly (p < 0.01) higher than the light hours (83.8 %) (Table 1). Likewise, large size *C. gariepinus* consumed all larvae (100 %) during night hours, which was a significantly (p < 0.01) higher rate compared to the light hours (78.6 %). There was no significant difference in the larval consumption by large size *C. gariepinus,* among the groups having 50, 100 and 150 larvae. The percentage of larval consumption was similar among the groups with the different amount of supplementary fish food.

**Table 1:** Predation of *Clarias gariepinus* on *Anopheles arabiensis* larvae during day light and day night hours

Fish size	Number exposed	Fish food	Light hour (From 6:00 am to 6:00 pm)		Night hour (from 6:00 pm to 6:00 am)		p values
			Average consumed (SE)	Percent consumed	Average consumed (SE)	Percent consumed	
Small	50	Adequate	41.7 (6.1)	83.3	46.7 (3.3)	93.3	0.119
	50	Inadequate	41.7 (4.4)	83.3	50.0 (0.0)	100.0	0.001
	50	No food	40.7 (2.3)	81.3	43.3 (6.7)	86.7	0.461
	100	Adequate	76.3 (11.6)	76.3	100.0 (0.0)	100.0	<0.001
	100	Inadequate	76.7 (8.8)	76.7	93.3 (6.7)	93.3	0.018
	100	No food	78.3 (6.0)	78.3	100.0 (0.0)	100.0	0.001
	150	Adequate	146.0 (1.2)	97.3	148.3 (1.7)	98.9	0.558
	150	Inadequate	135.3 (2.9)	90.2	150.0 (0.0)	100.0	0.023
	150	No food	130.0 (10.4)	86.7	150.0 (0.0)	100.0	0.008
Large	50	Adequate	45.0 (2.9)	90.0	50.0 (0.0)	100.0	0.022
	50	Inadequate	40.0 (2.9)	80.0	50.0 (0.0)	100.0	<0.001
	50	No food	41.0 (2.1)	82.0	50.0 (0.0)	100.0	0.002
	100	Adequate	79.0 (0.6)	79.0	100.0 (0.0)	100.0	0.001
	100	Inadequate	76.7 (1.7)	76.7	100.0 (0.0)	100.0	<0.001
	100	No food	76.7 (7.3)	76.7	100.0 (0.0)	100.0	<0.001
	150	Adequate	105.0 (2.9)	70.0	150.0 (0.0)	100.0	<0.001
	150	Inadequate	110.0 (2.9)	73.3	150.0 (0.0)	100.0	<0.001
	150	No food	120.0 (5.8)	80.0	150.0 (0.0)	100.0	0.001

Note: Average larvae consumed is the mean of the number of larvae consumed for three replicate experiments

*P values* significance test on the comparison of percent of *Anopheles arabiensis* larvae consumed between day light and day night hours

Small size *C. gariepinus* consumed larger number of *An. arabiensis* than the large size *C. gariepinus*. During the light hours, larval consumption by small size fish (83.7 %) was greater than the consumption by the large size fish (78.6 %). During the night hours, percentage of larvae consumed by large size *C. gariepinus* (100 %) was slightly greater than the percentage consumed by small size *C. gariepinus* (96.9 %).

### Feeding activity of *Clarias gariepinus* on culicine larvae under the laboratory condition

Culicine larvae consumption by small size (100 %) and large size (97.4 %) *C. gariepinus* during night hours was significantly (p < 0.01) greater compared to the corresponding consumption of 77.7 and 39.6 % during light hours (Table 2). Consumption of large size *C. gariepinus* decreased significantly (p < 0.01) with the increase in the number of culicine larvae per group during the day light hours.

**Table 2:** Predation of *Clarias gariepinus* on *Culex* mosquitoes during light and night hours

Fish size	Number exposed	Fish food	Light hour (From 6:00 am to 6:00 pm)		Night hour (from 6:00 pm to 6:00 am)		p value
			Average consumed (SE)	Percent consumed	Average consumed (SE)	Percent consumed	
Small	50	Adequate	25.0 (2.9)	50.0	50.0 (0.0)	100.0	<0.001
	50	Inadequate	40.0 (2.9)	80.0	50.0 (0.0)	100.0	0.001
	50	No	45.0 (2.9)	90.0	50.0 (0.0)	100.0	0.023
	100	Adequate	60.0 (8.7)	60.0	100.0 (0.0)	100.0	<0.001
	100	Inadequate	85.0 (2.9)	85.0	100.0 (0.0)	100.0	0.004
	100	No	80.0 (2.0)	80.0	100.0 (0.0)	100.0	0.001
	150	Adequate	146.0 (1.2)	97.3	150.0 (0.0)	100.0	0.217
	150	Inadequate	125.0 (2.9)	83.3	150.0 (0.0)	100.0	0.002
	150	No	110.0 (5.8)	73.3	150.0 (0.0)	100.0	<0.001
Large	50	Adequate	41.7 (4.4)	83.3	50.0 (0.0)	100.0	0.002
	50	Inadequate	25.0 (2.9)	50.0	43.3 (2.0)	86.7	<0.001
	50	No	23.3 (1.7)	46.7	50.0 (0.0)	100.0	<0.001
	100	Adequate	40.0 (2.9)	40.0	100.0 (0.0)	100.0	<0.001
	100	Inadequate	40.0 (2.9)	40.0	90.0 (2.9)	90.0	<0.001
	100	No	16.7 (4.4)	16.7	100.0 (0.0)	100.0	<0.001
	150	Adequate	40.0 (5.8)	26.7	150.0 (0.0)	100.0	<0.001
	150	Inadequate	50.0 (5.8)	33.3	150.0 (0.0)	100.0	<0.001
	150	No	30.0 (5.8)	20.0	150.0 (0.0)	100.0	<0.001

Note: Average larvae consumed is the mean of the number of larvae consumed for three replicate experiments

*P values* significance test on the comparison of percent of *Culex* larvae consumed between day light and day night hours

Percentage of culicine larvae consumed by small size *C. gariepinus,* during light hour, was significantly (p < 0.01) greater in the groups with no fish food (81.1 %) or inadequate fish food (82.8 %) compared to the groups with adequate food (69.1 %). However, the percentage of culicine larvae consumed by large size *C. gariepinus* during light hour was significantly (p < 0.01) greater in the groups with adequate (50 %) or inadequate (41.1 %) food compared to the groups with no food (27.8 %). On the other hand, the percentage of culicine larvae consumed by small and large *C. gariepinus* during night hours was similar among experiments with adequate, inadequate or no food at all and among experiments with different number of mosquito exposed (50, 100 and 150).

The percentage of culicine larvae consumed by small *C. gariepinus* during light (73.9 %) and night (100 %) hours was significantly greater than the percentages consumed by large size *C. gariepinus* during light (39.6 %) and night (97.4 %) hours, respectively (p < 0.01).

Large size *C. gariepinus* consumed greater number of *Anopheles* than culicine larvae during light (% consumed = 78.6 vs. 39.6, z = 9.72, p < 0.01) and night (% consumed = 100 vs. 97.4, p < 0.01) hours. Small size *C. gariepinus* consumed greater number of culicine than anopheline larvae during night hours (% consumed = 100 vs. 96.9, p < 0.01). The difference between the number of culicine and anopheline larvae consumed by small size *C. gariepinus* was not significant during light hours.

Small size *C. gariepinus* fish consumed greater number of mosquito larvae than the large size in the multivariable regression model (β = 13.36, 95 % CI = 4.57, 22.15) (Table 3). The number of *Anopheles* larvae consumed was greater than the number of culicine larvae consumed by the fish (β = 12.10, 95 % CI = 3.31, 20.89). In the model, the number of larvae consumed was greater during the night hours than during the light hours (β = 30.06, 95 % CI = 21.27, 38.85). The number of mosquito larvae consumed also increased significantly with the increase in the number of mosquito exposed. However, differences in the number of mosquito consumed were not significant among experiments where food was added at adequate or inadequate level or not at all. More than 80 % of *Anopheles* and *Culex* larvae placed in aquaria without *C. gariepinus* developed into adult mosquitoes within 2 weeks (Table 4).

**Table 3:** Effect of fish size, mosquito species, number of larvae exposed, supplementary food and hour of larval exposure on the predation of *Clarias gariepinus* on mosquito larvae

Variable	Average No. of larvae consumed	Crude mean difference in the Average No. of larvae consumed during light or night hours (95 % CI)	Adjusted mean difference in the Average Number of mosquito larvae during light or night hours (95 % CI)
Fish size			
Large	77.31	–	–
Small	90.67	13.36 (−6.57, 33.29)	13.36 (4.57, 22.15)
Mosquito			
* Culex*	77.94	–	–
* Anopheles*	90.04	12.10 (−7.87, 32.07)	12.10 (3.31, 20.89)
No. of larvae			
50	43.05	–	–
100	82.03	38.97 (24.35, 53.59)	38.97 (28.21, 49.74)
150	126.90	83.85 (69.22, 98.47)	83.85 (73.08, 94.61)
Fish food			
No food	82.71	–	–
Inadequate	84.25	1.54 (−23.35, 26.43)	1.54 (−9.22, 12.30)
Adequate	85.03	2.32 (−22.57, 27.21)	2.31 (−8.45, 13.08)
Hours of exposure			
Light (6:00 am–6:00 pm)	68.96	–	–
Night (6:00 pm–6:00 am)	99.03	30.06 (11.20, 48.93)	30.06 (21.27, 38.85)

Note: Average larvae consumed is the mean of the number of larvae consumed for three replicate experiments

**Table 4:** Emergence of adult *Anopheles* and *Culex* mosquito within two weeks after introduction of the larvae in aquaria without *Clarias gariepinus*

Larval type	Larval density	Number of adults emerged	% of adult mosquitoes emerged
Culicine larvae	50	42	84
	100	92	92
	150	147	98
*Anopheles* larvae	50	48	96
	100	89	89
	150	130	87

### Feeding activity of *Clarias gariepinus* on *An. arabiensis* under semi-field condition

Out of 300 *An. arabiensis* larvae placed in ponds where no catfish was introduced, 200 (67 %) emerged into adults within 7–14 days (Table 5). On the other hand, in the other 3 experimental groups, where small, medium or large size catfish and 300 *Anopheles* larvae were added, no adult mosquito emerged.

**Table 5:** Feeding activity of *Clarias gariepinus* against *Anopheles arabiensis* larvae (n = 300 per group) in semi-field experiment, May 2013

Treatment groups	Supplementary food	Larvae consumed	% of dead larvae or adults	% of adults mosquito emerged	Time taken
Large fish	Inadequate	All	–	–	3 days
Medium fish	Inadequate	All	–	–	3 days
Small fish	Inadequate	All	–	–	3 days
No fish	–	–	33	67	7–14 days

## Discussion

African catfish, *Clarias gariepinus,* was observed to feed on the larvae of *Anopheles arabiensis* and culicine mosquitoes under laboratory and semi-field conditions. The fish consumed the larvae in the presence or absence of supplementary fish food and also with different larval density. Larval consumption was significantly correlated with the size of the fish, mosquito genera, number of mosquito initially exposed and period of exposure (day light or day night hours).

*C. gariepinus* consumed greater number of larvae during the night (from 6:00 pm to 6:00 am) than during the light hours. The larval feeding activity of *C. gariepinus* was reported to be higher in dark hours than in the light hours, or alternating light and dark conditions [12, 13]. The feeding activity of the fish was found to be affected by its sensory organs rather than its visual sense organs [14, 15]. The test buds and free neurocytes are common in the fish; hence the fish gets active to feed in dark conditions. In addition, the activities of mosquito larvae increase at night hours [15, 16], making them easily exposed to the fish.

Percentage of larval consumption was greater in experiments that involved small size *C. gariepinus* than those with the large size. Small size fish are at a lower risk of recognition by predators and usually remain active in day and night hours. These attributes make them better and potent control agents for mosquito larvae. Larvivorous fish which are small, hardy and capable of getting about easily in shallow waters among thick weeds are usually preferred to control mosquito larvae. Crustaceans, insect larvae, small vertebrates, invertebrates and young fishes make up the diet of small *C. gariepinus* [17]. Furthermore, young fish may also have higher metabolic rates compared to large adult fish, necessitating more intake of larvae and other food. On the other hand, large size *C. gariepinus* prefers to feed more on larger prey compared with small ones [18, 19].

*C. gariepinus* consumed greater number of *An. arabiensis* larvae than culicine. This could be due to the lower motility rate and water surface resting behavior of *An. arabiensis* larvae, which exposes them for easy detection by the fish. On the other hand, culicine mosquito larvae make frequent mobility in water bodies thereby avoiding easy capture by the fish. *C. gariepinus* detects food with its sensory barbells before securing with its teeth and gulping, thus *C. gariepinus* prefer inactive preys [17, 20].

There was significant correlation between the number of mosquito larvae consumed and the number of mosquito larvae initially exposed. Larval consumption was higher in groups with large number of mosquitoes (e.g. n = 150) than in groups with small numbers (n = 100 or n = 50). However, the number of mosquito larvae consumed was similar among the groups with the different amount of supplementary fish food.

In some of the laboratory control experiments where mosquito of different larval density were introduced into an aquaria without *C. gariepinus*, 90 % of the mosquito larvae added developed into adults. Similarly, in pond experiments, about 66 % of mosquito larvae placed in aquaria without *C. gariepinus* developed into adults. On the other hand, all the mosquito larvae in the experimental groups in which *C. gariepinus* introduced were consumed by the fish. This finding was in agreement with the previous experimental evidence for mosquito larva consumption by the catfish [21].

*C. gariepinus* can endure extremely harsh conditions and able to tolerate very low oxygen rmis concentrations allowing ease of local production and transportation of the fish [14, 20]. In addition, the habitat of *C. gariepinus* overlaps with those of mosquito larvae [11]. The observation that *C*. *gariepinus* feeds on *Biomphalaria pfeifferi* (the intermediate host of *Schistosoma mansoni*) [11] also makes this fish a good candidate for biological control agent against the mosquitoes and snail intermediate hosts as their habitats overlap in many cases. Nevertheless, introduction of alien fish species into a natural ecosystem as a biocontrol agent could threaten native fish species and other aquatic biota through introduction of disease causing pathogens, predatory and biological competition [22].

## Conclusions

The greater percentage of *Anopheles* and culicine larvae were consumed when introduced to aquaria containing *C. gariepinus* during day and night hours. The feeding efficacy of *C. gariepinus* showed significant correlation with the size of the fish, hours of feeding, mosquito species and number exposed. However, further studies about the feeding efficacy of the fish in pools and ponds and long term environmental effects of *C. gariepinus* on other aquatic species should be carefully examined before recommending its introduction for biological control of mosquitoes.

## Abbreviations

IRS: indoor residual spraying
LLITNs: long-lasting insecticide treated nets
NFLARRC: National Fisheries and Other Living Aquatic Resources Research Centre
ALIPB: Aklilu Lemma Institute of Pathobiology
CI: confidence interval
